# A monoclinic polymorph of cysteamine hydro­chloride

**DOI:** 10.1107/S1600536809053008

**Published:** 2009-12-12

**Authors:** Saeed Ahmad, Muhammad Ashraf Shaheen, Helen Stoeckli-Evans

**Affiliations:** a Department of Chemistry, University of Engineering and Technology, Lahore 54890, Pakistan; b Department of Chemistry, University of Sargodha, Sargodha, Pakistan; c Institute of Physics, University of Neuchâtel, rue Emile Atgand 11, CH-2009 Neuchâtel, Switzerland

## Abstract

The title compound (systematic name: 2-mercaptoethan­aminium chloride), C_2_H_8_NS^+^·Cl^−^, the hydro­chloride salt of cysteamine, in contrast to the previously reported triclinic polymorph [Kim *et al.* (2002 ▶). *Polyhedron*, **21**, 225–228], crystallized in the monoclinic crystal system. In the crystal, the cysteaminium cations are linked to the chloride anions *via* one S—H⋯Cl and three N—H⋯Cl hydrogen bonds. Two-dimensional slab-like networks are formed, which are stacked in [100]. This arrangement is similar to that observed in the triclinic polymorph.

## Related literature

For the structure of the triclinic polymorph, see: Kim *et al.* (2002 ▶).
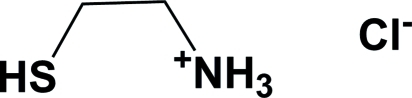


## Experimental

### 

#### Crystal data

C_2_H_8_NS^+^·Cl^−^
*M* *_r_* = 113.60
Monoclinic, 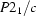

*a* = 7.7441 (4) Å
*b* = 8.4931 (5) Å
*c* = 8.7126 (5) Å
β = 101.962 (4)°
*V* = 560.60 (5) Å^3^
*Z* = 4
Mo *K*α radiation
μ = 0.90 mm^−1^
*T* = 173 K
0.40 × 0.40 × 0.40 mm

#### Data collection

Stoe IPDS-2 diffractometer
Absorption correction: numerical (*X-SHAPE*; Stoe & Cie, 2009 ▶) *T*_min_ = 0.738, *T*_max_ = 0.860
10581 measured reflections
1506 independent reflections
1426 reflections with *I* > 2σ(*I*)
*R*_int_ = 0.072

#### Refinement

*R*[*F* ^2^ > 2σ(*F* ^2^)] = 0.032
*wR*(*F* ^2^) = 0.088
*S* = 1.10
1506 reflections
79 parameters
All H-atom parameters refined
Δρ_max_ = 0.30 e Å^−3^
Δρ_min_ = −0.36 e Å^−3^

### 

Data collection: *X-AREA* (Stoe & Cie, 2009 ▶); cell refinement: *X-AREA*; data reduction: *X-RED32* (Stoe & Cie, 2009 ▶); program(s) used to solve structure: *SHELXS97* (Sheldrick, 2008 ▶); program(s) used to refine structure: *SHELXL97* (Sheldrick, 2008 ▶); molecular graphics: *ORTEP-3* (Farrugia, 1997 ▶) and *Mercury* (Macrae *et al.*, 2006 ▶); software used to prepare material for publication: *SHELXL97* and *PLATON* (Spek, 2009 ▶).

## Supplementary Material

Crystal structure: contains datablocks I, global. DOI: 10.1107/S1600536809053008/is2503sup1.cif

Structure factors: contains datablocks I. DOI: 10.1107/S1600536809053008/is2503Isup2.hkl

Additional supplementary materials:  crystallographic information; 3D view; checkCIF report

## Figures and Tables

**Table 1:** Hydrogen-bond geometry (Å, °)

*D*—H⋯*A*	*D*—H	H⋯*A*	*D*⋯*A*	*D*—H⋯*A*
S1—H1*S*⋯Cl1^i^	1.21 (3)	2.69 (3)	3.8003 (5)	152 (2)
N1—H1*AN*⋯Cl1^ii^	0.89 (3)	2.31 (3)	3.1485 (13)	159 (2)
N1—H1*BN*⋯Cl1^iii^	0.89 (2)	2.44 (2)	3.2563 (14)	152 (2)
N1—H1*CN*⋯Cl1	0.90 (3)	2.26 (3)	3.1437 (13)	169 (2)

Symmetry codes: (i) 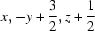; (ii) 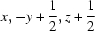; (iii) 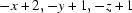.

**Fig. 1.**
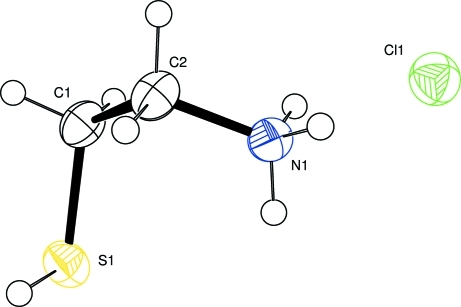
A view of the molecular structure of the title compound, with the displacement ellipsoids drawn at the 50% probability level.

**Fig. 2.**
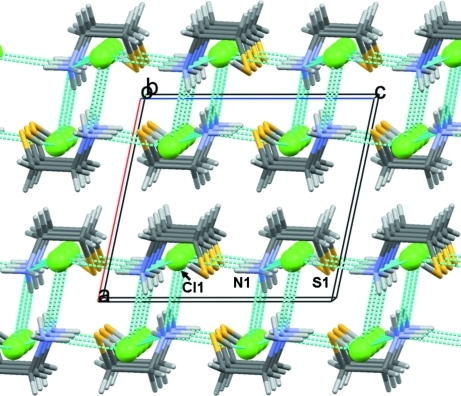
A view, along the b axis, of the crystal packing of the title compound. The S—H···Cl and N—H···Cl hydrogen bonds are shown as dotted cyan lines (see Table 1 for details).

**Fig. 3.**
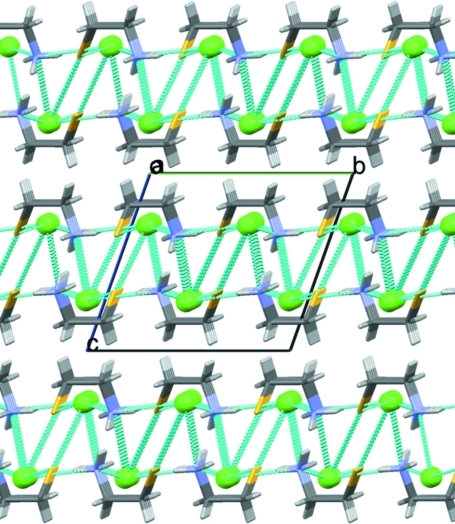
A view, along the a axis, of the crystal packing in the triclinic polymorph of the title compound (Kim et al., 2002). The S—H···Cl and N—H···Cl hydrogen bonds are shown as dotted cyan lines.

## supplementary crystallographic information

### Comment

The crystal structure of the triclinic polymorph, (I), of the title compound has been reported previously (Kim *et al.*, 2002). Those crystals were prepared by recrystallization of cysteamine hydrochloride from hot alchohols, such as n-butanol, 2-propanol or n-propanol.

The stucture of the monoclinic polymorph, (II), is illustrated in Fig. 1, and the geometrical parameters are available in the Supplementary Information and the archived CIF. Here the crystalline sample received from the producers was used without further recrystallization. In contrast to (I), that crystallized with two independent molecules per asymmetric unit, polymorph (II) crystallized with one independent molecule per asymmetric unit. The conformation of the cation (*i.e.* torsion angle S—C—C—N) is similar in the two polymorphs: 61.49 (16)° in (II), and -60.28 and 60.65° in (I).

In the crystal of (II) the cysteaminium cations are linked to the chloride anions, *via* one S—H···Cl and three N—H···Cl hydrogen bonds (Table 1). Two-dimensional slab-like networks are formed, which stack in the [100] direction (Fig. 2). A similar hydrogen-bonded slab-like arrangement was also observed in the crystal structure of the triclinic polymorph (I), see Fig. 3.

### Experimental

The sample used, supplied by Alfa Aesar (A Johnson Matthey Company) USA, consisted of colourless block-like crystals. A small piece of a large crystal was used for data collection.

### Refinement

The H-atoms were all located in a difference electron-density map and were freely refined: S—H = 1.21 (3) Å; N—H = 0.89 (3)–0.90 (3) Å; C—H = 0.95 (2)–0.991 (17) Å.

### Crystal data

**Table:**

C_2_H_8_NS^+^·Cl^−^	*F*(000) = 240
*M**_r_* = 113.60	*D*_x_ = 1.346 Mg m^−^^3^
Monoclinic, *P*2_1_/*c*	Mo *K*α radiation, λ = 0.71073 Å
Hall symbol: -P 2ybc	Cell parameters from 15168 reflections
*a* = 7.7441 (4) Å	θ = 2.4–29.5°
*b* = 8.4931 (5) Å	µ = 0.90 mm^−^^1^
*c* = 8.7126 (5) Å	*T* = 173 K
β = 101.962 (4)°	Block, colourless
*V* = 560.60 (5) Å^3^	0.40 × 0.40 × 0.40 mm
*Z* = 4	

### Data collection

**Table:**

Stoe IPDS-2 diffractometer	1506 independent reflections
Radiation source: fine-focus sealed tube	1426 reflections with *I* > 2σ(*I*)
graphite	*R*_int_ = 0.072
φ and ω scans	θ_max_ = 29.2°, θ_min_ = 2.7°
Absorption correction: numerical (*X-SHAPE*; Stoe & Cie, 2009)	*h* = −10→10
*T*_min_ = 0.738, *T*_max_ = 0.860	*k* = −11→11
10581 measured reflections	*l* = −11→11

### Refinement

**Table:**

Refinement on *F*^2^	Secondary atom site location: difference Fourier map
Least-squares matrix: full	Hydrogen site location: inferred from neighbouring sites
*R*[*F*^2^ > 2σ(*F*^2^)] = 0.032	All H-atom parameters refined
*wR*(*F*^2^) = 0.088	*w* = 1/[σ^2^(*F*_o_^2^) + (0.0466*P*)^2^ + 0.1619*P*] where *P* = (*F*_o_^2^ + 2*F*_c_^2^)/3
*S* = 1.10	(Δ/σ)_max_ = 0.001
1506 reflections	Δρ_max_ = 0.30 e Å^−^^3^
79 parameters	Δρ_min_ = −0.36 e Å^−^^3^
0 restraints	Extinction correction: *SHELXL97* (Sheldrick, 2008), Fc^*^=kFc[1+0.001xFc^2^λ^3^/sin(2θ)]^-1/4^
Primary atom site location: structure-invariant direct methods	Extinction coefficient: 0.038 (9)

### Special details

**Table:**

Geometry. Bond distances, angles *etc*. have been calculated using the rounded fractional coordinates. All su's are estimated from the variances of the (full) variance-covariance matrix. The cell e.s.d.'s are taken into account in the estimation of distances, angles and torsion angles
Refinement. Refinement of *F*^2^ against ALL reflections. The weighted *R*-factor *wR* and goodness of fit *S* are based on *F*^2^, conventional *R*-factors *R* are based on *F*, with *F* set to zero for negative *F*^2^. The threshold expression of *F*^2^ > σ(*F*^2^) is used only for calculating *R*-factors(gt) *etc*. and is not relevant to the choice of reflections for refinement. *R*-factors based on *F*^2^ are statistically about twice as large as those based on *F*, and *R*- factors based on ALL data will be even larger.

### Fractional atomic coordinates and isotropic or equivalent isotropic displacement parameters (Å^2^)

**Table:**

	*x*	*y*	*z*	*U*_iso_*/*U*_eq_	
S1	0.85152 (6)	0.67255 (4)	0.96395 (5)	0.0370 (1)	
N1	0.83799 (17)	0.42811 (15)	0.67906 (15)	0.0288 (3)	
C1	0.6807 (2)	0.53275 (19)	0.87903 (17)	0.0327 (4)	
C2	0.67171 (19)	0.4989 (2)	0.70672 (17)	0.0326 (4)	
Cl1	0.77972 (4)	0.40823 (4)	0.31160 (4)	0.0290 (1)	
H1AN	0.853 (3)	0.335 (3)	0.726 (3)	0.045 (6)*	
H1A	0.711 (3)	0.439 (3)	0.942 (3)	0.038 (5)*	
H1B	0.570 (3)	0.572 (3)	0.894 (3)	0.045 (6)*	
H1S	0.795 (3)	0.783 (3)	0.881 (3)	0.060 (7)*	
H1BN	0.935 (3)	0.485 (3)	0.714 (3)	0.044 (6)*	
H2A	0.652 (3)	0.597 (2)	0.644 (2)	0.033 (5)*	
H2B	0.575 (3)	0.428 (3)	0.668 (3)	0.055 (7)*	
H1CN	0.834 (3)	0.414 (3)	0.576 (3)	0.047 (6)*	

### Atomic displacement parameters (Å^2^)

**Table:**

	*U*^11^	*U*^22^	*U*^33^	*U*^12^	*U*^13^	*U*^23^
S1	0.0457 (3)	0.0318 (2)	0.0344 (2)	−0.0024 (1)	0.0107 (2)	−0.0034 (1)
N1	0.0285 (6)	0.0321 (6)	0.0256 (6)	−0.0013 (4)	0.0053 (4)	0.0005 (5)
C1	0.0301 (7)	0.0407 (8)	0.0284 (7)	0.0010 (6)	0.0083 (5)	0.0038 (6)
C2	0.0273 (6)	0.0432 (8)	0.0265 (6)	0.0019 (6)	0.0041 (5)	0.0032 (6)
Cl1	0.0289 (2)	0.0307 (2)	0.0268 (2)	0.0011 (1)	0.0046 (1)	−0.0016 (1)

### Geometric parameters (Å, °)

**Table:**

S1—C1	1.8170 (16)	C1—C2	1.516 (2)
S1—H1S	1.21 (3)	C1—H1A	0.97 (3)
N1—C2	1.485 (2)	C1—H1B	0.95 (2)
N1—H1BN	0.89 (2)	C2—H2A	0.991 (17)
N1—H1AN	0.89 (3)	C2—H2B	0.97 (2)
N1—H1CN	0.90 (3)		
			
C1—S1—H1S	96.9 (12)	S1—C1—H1B	108.4 (15)
H1AN—N1—H1CN	108 (2)	C2—C1—H1A	111.2 (15)
H1BN—N1—H1CN	105 (2)	C2—C1—H1B	110.1 (15)
C2—N1—H1AN	108.7 (16)	H1A—C1—H1B	109 (2)
C2—N1—H1BN	115.0 (16)	N1—C2—H2A	106.9 (13)
C2—N1—H1CN	111.4 (15)	N1—C2—H2B	109.1 (15)
H1AN—N1—H1BN	108 (2)	C1—C2—H2A	110.9 (10)
S1—C1—C2	114.04 (11)	C1—C2—H2B	109.7 (15)
N1—C2—C1	111.96 (12)	H2A—C2—H2B	108 (2)
S1—C1—H1A	103.7 (15)		
			
S1—C1—C2—N1	61.49 (16)		

### Hydrogen-bond geometry (Å, °)

**Table:**

*D*—H···*A*	*D*—H	H···*A*	*D*···*A*	*D*—H···*A*
S1—H1S···Cl1^i^	1.21 (3)	2.69 (3)	3.8003 (5)	152 (2)
N1—H1AN···Cl1^ii^	0.89 (3)	2.31 (3)	3.1485 (13)	159 (2)
N1—H1BN···Cl1^iii^	0.89 (2)	2.44 (2)	3.2563 (14)	152 (2)
N1—H1CN···Cl1	0.90 (3)	2.26 (3)	3.1437 (13)	169 (2)

Symmetry codes: (i) *x*, −*y*+3/2, *z*+1/2; (ii) *x*, −*y*+1/2, *z*+1/2; (iii) −*x*+2, −*y*+1, −*z*+1.
